# Ex vivo evaluation of the accuracy of four electronic foraminal locators during endodontic retreatment 

**DOI:** 10.4317/jced.61133

**Published:** 2024-03-01

**Authors:** Ilana-Thais-de Freitas Lima, Ana-Letícia-Linhares-de Sousa Paula, Reuton-dos Santos Palheta-Filho, Amanda-Brito Santos, Nathalia-de Aguiar Freitas, George-Táccio-de Miranda Candeiro

**Affiliations:** 1 MSC, Department of Dentistry, Unichristus, Fortaleza, Ceará, Brazil; 2 DDS, Department of Dentistry, Unichristus, Fortaleza, Ceará, Brazil; 3 Department of Dentistry, Unichristus, Fortaleza, Ceará, Brazil; 4 PhD, Department of Dentistry, Unichristus, Fortaleza, Ceará, Brazil

## Abstract

The present study aimed to evaluate the accuracy of four different Electronic Foraminal Locators (EFLs): Root ZX II (J. Morita, Tokyo, Japan), RomiApex A15 (Romidan, Kiryat-Ono, Israel), FinePex (Schuster, Porto Alegre, Brazil) and VDW Gold (VDW, Munich, Germany), in determining root length during endodontic retreatment steps. Twenty-seven human unirooted teeth had their crowns sectioned to standardize the teeth to 17 mm. The actual tooth length was visualized with an operating microscope and a #15 file juxtaposed to the apical foramen. Teeth were instrumented with files R25 and R40, and at the end of each instrumentation, measurements of root canal lengths were made with files #25 and #40. Then, the teeth had their root canals filled with standardized Gutta-Percha R40 cones and Endofill cement, and after seven days, they were uncovered with R25 and R40 files, respectively. New measurements were made with #25 and #40 files between the uncovering with each file. The data were statistically analyzed by ANOVA and Chi-square tests, considered significant when *P*<0.05. All devices tended to under-measurement when the obturating material was partially removed with the R25 file. When the canals were uncovered with the R40 instrument, the effectiveness of the appliances increased significantly (*P*<0.05). At 0.40 mm diameter, the mean accuracy of the Romiapex A15 appliance was statistically lower than the other EFLs (*P*<0.001), showing a tendency to over-measurement. In conclusion, all the tested appliances showed similar efficacy when acceptable limits were observed. The permanence of the remaining filling material in the apical third influenced the accuracy and efficacy of EFLs in endodontic retreatment cases.

** Key words:**Endodontics, odontometry, apical foramen.

## Introduction

Endodontic retreatment is the first alternative for reintervention in cases of initial therapy failure and has success rates of approximately 75% (1,2). One of the factors favoring successful therapy is the accurate and thorough cleaning of the root canal system without harming apical integrity (3). However, secondary infections, persistent infections, or failure of the initial endodontic treatment compromise the tooth, leading to retreatment (4).

The endodontic retreatment procedure initially requires complete root canal desobturation, chemical-surgical preparation (CSP), and obturation. Accurate determination of the root canal length is of utmost importance to limit instrumentation and obturation within the boundaries of the root canal (5). However, one of the most challenging steps of endodontic retreatment is completely removing the root canal filling material. Several previous works have shown the difficulty of removing gutta-percha and endodontic cement, regardless of the manual or mechanized instrumentation system (6-8). For this, additional procedures, such as ultrasonic activations, can be used to optimize the removal (8).

Electronic CT determination utilizing EFLs is the safest method under ideal conditions and has an accuracy above 95% 9. EFLs are useful for locating the apical foramen (AF) and measuring root length (5,10).

However, during retreatment, the correct determination of the CT may present a challenge, Wilcox *et al*. (11), report that it is almost impossible to completely remove filling materials from the root canal walls. The accuracy of EFLs in determining CT after root canal desobturation is not yet fully understood (12). However, Alves *et al*. (13), observed that residual gutta-percha inside the canal prevented signal transmission until the instrument bypassed the filling material. In their study, the root canal lengths determined by EFLs were greater at retreatment. 

To date, few studies have evaluated the accuracy of EFLs during endodontic retreatment. However, no study has evaluated the accuracy of EFLs: Root ZX II, RomiApex A15, FinePex, and VDW Gold. Thus, the present study aimed to analyze the accuracy of four different EFLs (Root ZX II, RomiApex A15, FinePex, and VDW Gold) during endodontic retreatment and whether the remaining amount of filling material influences the accuracy of these appliances.

## Material and Methods

Initially, the present study was approved by the Research Ethics Committee of the Christus University Center with opinion number 3.099.081.

Twenty-seven human unirooted teeth were selected for this study, fitting the following inclusion criteria: teeth with fully formed apices, with an apical diameter smaller than 0.25 mm, without resorption, calcification, or dilaceration, and previous endodontic treatment. The teeth were kept immersed in saline solution until the moment of use.

The crowns of the teeth were sectioned, standardizing the root length at 17 mm, and flattened to facilitate the positioning of the rubber cursor during measurement, thus favoring measurement accuracy. For this purpose, a diamond disk (KG Sorensen, Cotia, Brazil) and a digital caliper (Mitutoyo, Suzano, Brazil) were required. After standardizing the specimens, coronary access was performed with diamond tip no. 1013 and no. 3081 (KG Sorensen, Cotia, Brazil) on teeth that remained with an integral crown. The root canals were explored with manual file K#10 (Dentsply Maillefer, Ballaigues, Switzerland).

The actual tooth length (ATL) was obtained by visualization with an operating microscope (Alliance, São Carlos, Brazil) at 10x magnification and K#15 file (Dentsply Maillefer, Ballaigues, Switzerland) juxtaposed to the apical foramen (AF). The file was measured with a digital caliper (Mitutoyo, Suzano, Brazil). The ATL was used as a reference for the other measurements (Reading 1).

The cervical and middle thirds were prepared with a CP Drill (Helse Dental Technology, São Paulo, Brazil). The teeth were attached to an acrylic device and immersed in a medium (Carbopol Gel, 0.9% NaCl, and 2% KCl - Farma Vie Farmácia de Manipulação, Fortaleza, Brazil).

The chemical-surgical preparation (CSP) was performed in the ATL (Reading 1) with R25 and R40 files, coupled to the VDW Gold motor (VDW, Munich, Germany) under saline solution irrigation. The canals were dried with EndoFlex endodontic suction (Maquira, Maringá, Paraná, Brazil) and Reciproc R40 absorbent paper tip (VDW, Munich, Germany). The teeth had their root canals filled with standardized Gutta-Percha cone Reciproc VDW R40 (VDW, Munich, Germany) and Endofill endodontic cement (Dentsply Maillefer, Ballaigues, Switzerland).

The specimens were kept for seven days in humidity inside an oven at 37º. After this period, the root canals were uncovered in CT (ATL-1mm) with R25 files, driven by VDW Gold motor, and irrigated with saline solution in a 5ml irrigation syringe with luer lock thread, and NaviTip irrigation cannula (Ultradent, Indaiatuba, São Paulo), followed by removal of the excess irrigation solution with EndoFlex endodontic sucker (Maquira, Maringá, Paraná).

Reading 2 was done using hand file K#25, digital caliper, and the EFLs Root ZX II (J Morita, Tokyo, Japan), RomiApex A15 (Romidan, Kiryat-Ono, Israel), FinePex (Schuster, Porto Alegre, Brazil) and VDW Gold motor (VDW, Munich, Germany). The measurements were made in triplicate to obtain the average between the measurements. The file was inserted slowly into the root canal until the “OVER” signal was obtained, and then the instrument was retracted until the “APEX” or “0.0” signal was reached. After five seconds of stability, the silicone cursor of the file was positioned, and the file was measured with a digital caliper. The measurements were recorded in Microsoft Excel spreadsheets.

**Figure 1:**
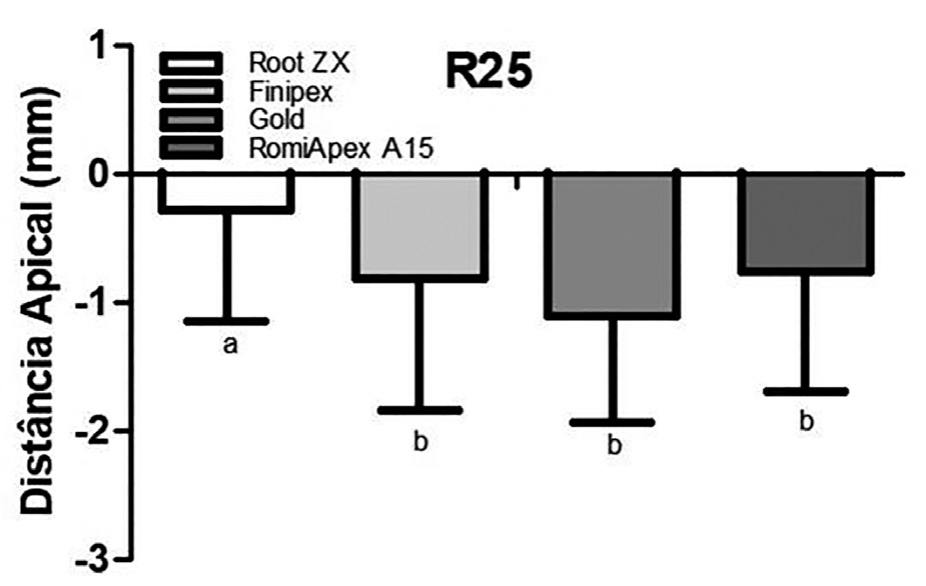
Means and standard deviation of the apical distance obtained by four different systems after debonding with the R25 file.

Next, the canals were prepared with an R40 file and irrigated with saline solution. Reading 3 was performed similarly to the previous reading; the K#40 file was employed for measurement. Figure 1 shows an organizational chart of the experimental steps.

After measurements were tabulated and spreadsheets of the software Microsoft Excel, the quantitative data were subjected to the Kolmogorov-Smirnov normality test, expressed as mean and standard deviation, and analyzed by ANOVA test followed by the Bonferroni post-test. Then, the measurements were categorized based on the clinical relevance of measuring the size of the dental unit between - 0.50 and + 0.50, between -1.00 and + 1.00, and between -1.50 and + 1.50. Data were expressed as absolute and percentage frequency and analyzed by the chi-square test. The significance level of the study was set at 5%.

## Results

In retreatment cases, it was observed that all appliances tended to under-measurement when the canals were partially uncovered with the R25 instrument (Table 1, Fig. 1). The mean accuracy of the Root ZX II appliance was statistically higher than the other EFLs (*P*<0.001). Table 2 shows the values of the measurements observed in each measurement, divided by distances from the apical foramen after desobturation of the R25 instrument.

**Table 1:**
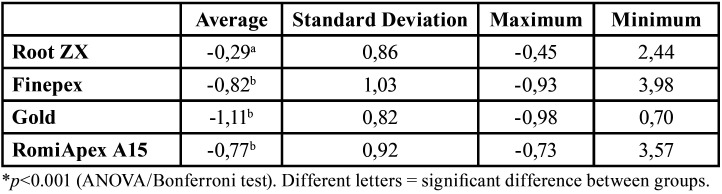
Means and standard deviation of the apical distance obtained by four different systems after debonding with the R25 file.

**Table 2:**
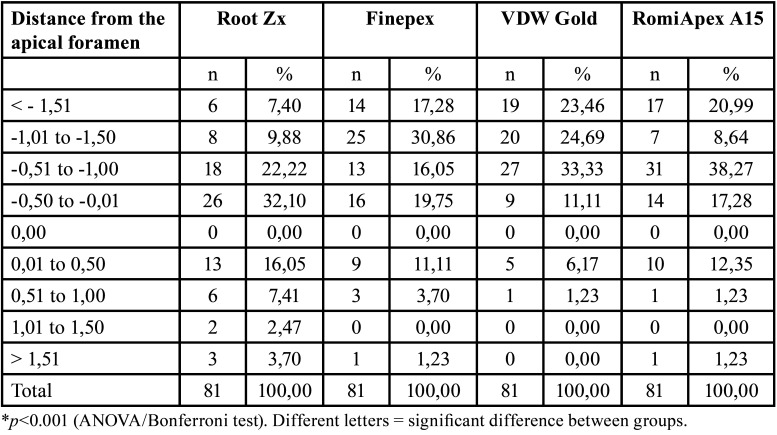
Analysis by distances of the apical foramen (measurements with 25.02) after retreatment with the R25 instrument.

**Figure 2:**
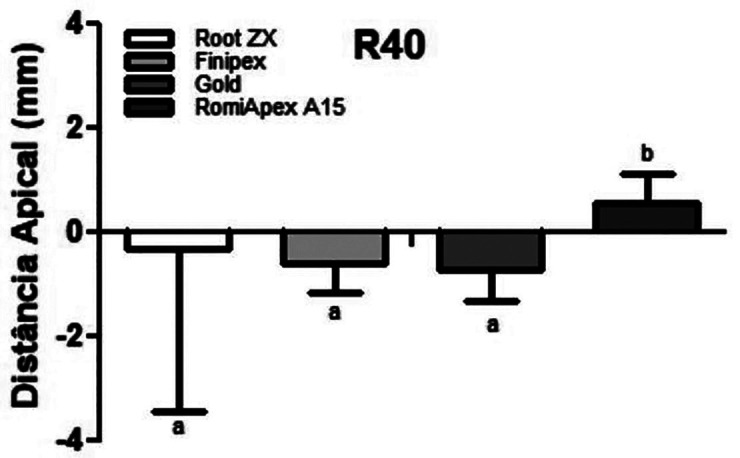
Means and standard deviation of the apical distance obtained by four different systems after debonding with the R40 file.

After desobturation with 0.40 mm diameter instruments (R40), the mean accuracy of the Romiapex A15 appliance was statistically lower than the other EFLs (*P*<0.001). However, only the Romiapex A15 appliance tended to over-measurements (Table 3, Fig. 2). Table 4 shows the values of the measurements observed in each measurement, divided by distances from the apical foramen after desobturation of the R40 instrument.

**Table 3:**
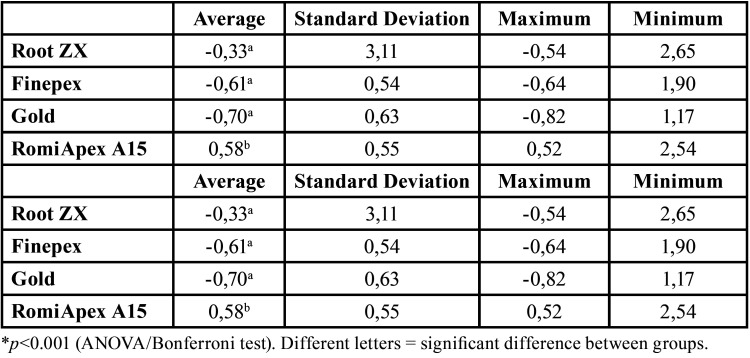
Means and standard deviation of the apical distance obtained by four different systems after debonding with the R40 file.

**Table 4:**
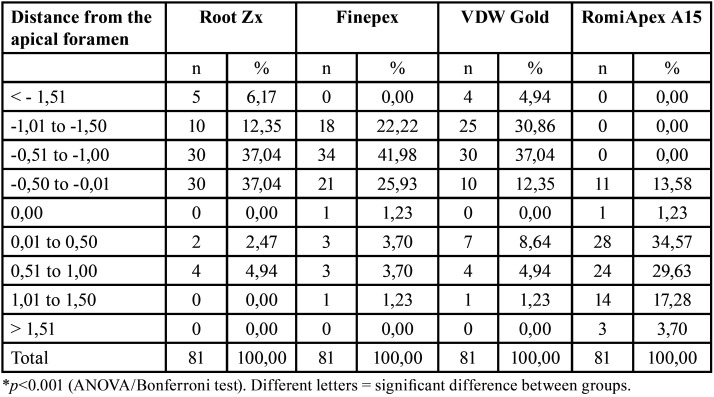
Analysis by distances of the apical foramen (measurements with 40.02) after retreatment with the R40 instrument.

It was observed that all appliances showed a tendency to under-measurements when canals were removed with R25 instruments only (Table 2). When the canals were removed with R40 instruments, the Root ZX II and Finepex appliances decreased the frequency of overmedications, while the Romiapex A15 appliance showed a significant increase in overmedications, respectively (Table 4).

Regarding the accepTable limits of variation, it was observed that the instruments showed similar efficiencies in determining odontometry (*P*>0.05), both when the R25 and R40 instruments were removed (Table 5). The measurements made on the appliances after uncovering with the R25 file were worse than those made with the R40 instrument. For all the instruments, the measurement efficiency was directly proportional to the increase of the accepTable variation limit, with efficiencies ranging from 17.28% to 100%, depending on the limit to be considered. The Finepex, VDW Gold, and Romiapex A15 devices showed significant changes in the means of the readings when the diameter of the apical instrument increased (*P*<0.001).

**Table 5:**
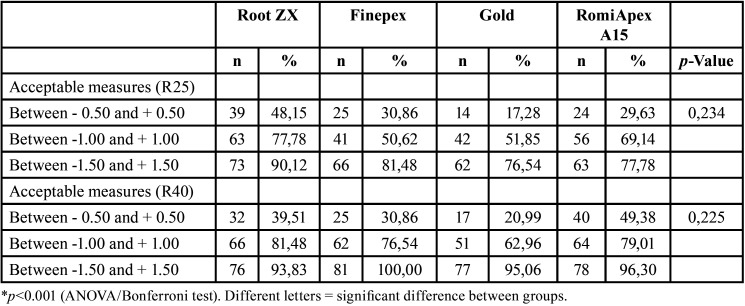
Absolute frequency and percentage of the apical distance levels obtained by four different systems after retreatment with R25 and R40 files.

## Discussion

As with initial endodontic treatment, CSP at the proper limits is essential for completely removing bacteria and necrotic tissue. Thus, using EFLs is currently indispensable and adequate for obtaining the working length 3,10. Previous works have shown the importance of this technology due to its greater accuracy when compared to traditional radiographic methods, also decreasing the radiation dose administered to the patient 3. However, the accuracy of these devices is still not fully clarified during endodontic retreatment.

During retreatment, root canal desobturation is an important step that should be performed with caution to allow disinfection of the root canals (14). However, completely removing filling materials adhered to the canal walls represents a major challenge (13). The presence of these remaining materials adhering to the canal walls may cause retreatment failure (12). Therefore, efforts must be made to ensure complete root canal debridement during endodontic retreatment.

In endodontic retreatment, a vital factor for success is establishing a correct apical working limit, where the root canal should be cleaned and shaped. Thus, EFLs are essential tools that safely and accurately determine the working limits during endodontic treatment. However, Al Balushi *et al*. (15) reported that the presence of endodontic filling materials might influence the impedance of root canals, and consequently, the effectiveness of EFLs may be impaired in cases of endodontic retreatment.

Aggarwal *et al*. (5) reported that EFLs are excellent adjuncts in determining root canal measurements, but they cannot replace radiographs. They should be used cautiously because there are chances of over-instrumentation in cases of extrusion of the filling material. Mancini *et al*. (16) consider that odontometry performed with LEF during endodontic retreatment can lead the root canal preparation beyond the apical foramen and consequent overobturation. In contrast, Ebrahim *et al*. (12) state that EFLs provide reliable measurements during retreatment.

The results of this study show that the devices tended to under-measurement when the 0.25 mm diameter (instrument R25) was used to promote desobturation, keeping part of the apical filling material. Furthermore, it was observed that only the RomiApex A15 appliance showed significant over-measurements when debonding occurred with 0.40 mm diameter instruments (R40), with little or no root filling material in the apical third. Based on the study of Shabahang *et al*. (17), which considers the measurement of ± 1 mm from the apical foramen clinically accepTable, the results presented in this study are pretty significant. Nekoofar *et al*. (18) consider the anatomical limit at 0.5 mm and 1 mm from the apical foramen as the ideal limit for instrumentation and obturation.

Corroborating with the present study, Ustun *et al*. (19) found differences between the ATL measurements and the Tri Auto ZX electronic measurement (J. Morita, Tokyo, Japan) in manual mode. The electronic measurements presented short of the apical foramen during endodontic retreatment.

When observing the limits of accepTable measurements, all devices showed similar efficacy. Similarly, Goldberg *et al*. (20) evaluated the accuracy of three different EFLs during the retreatment process and found no statistically significant differences between the devices. The readings obtained with the devices tested showed an accuracy of 95-100%. In the present study, only when the limit between ± 1.5 mm was considered, and with the desobturation occurring with the instrument with the largest diameter, was the efficiency of the appliances above 90%.

Unlike the results of Mancini *et al*. (16), who observed over-measurements in odontometries performed with EFLs after desobturation and statistically significant differences between ATL and the electronic measurements after endodontic treatment and retreatment. In agreement with the results obtained in previous studies, EFLs have been observed to provide reliable measurements during retreatment and have high accuracy rates (5,12,19,20).

Given this result, it can be seen that using EFLs is important for successful endodontic retreatment, and the clinician should be willing to remove the filling material completely to increase the accuracy and efficacy of EFLs.

## Conclusions

The remaining filling material in the apical third influenced the odontometry measurement using EFLs.

The accuracy of the appliances was inversely proportional to the amount of apical filling material.

The Root ZX II appliance showed the highest stability in odontometric readings with EFLs.

The devices showed similar efficacy when the limits of acceptable measurements were observed.

All devices tended to under-measurement when canals were uncovered with instruments smaller than the apical obturation diameter.

Only the Romiapex A15 appliance showed a greater tendency to oversize when the canals were uncovered with instruments of equal diameter to the apical diameter of the filling.
